# Crafting the Future of Digitization: How and When Digital Leadership Promotes Public Employees’ Proactive Service Performance

**DOI:** 10.3390/bs16061035

**Published:** 2026-06-21

**Authors:** Shanghao Song, Chenhui Zuo, Yunsheng Shi, Shujie Chen, Jingwei Zhao

**Affiliations:** 1 School of Labor Economics, Capital University of Economics and Business, Beijing 100070, China; shanghao.song@cueb.edu.cn (S.S.); chenshujie@cueb.edu.cn (S.C.); 2 School of Government, Beijing Normal University, Beijing 100875, China; zuochenhui@mail.bnu.edu.cn; 3 School of Marxism, Hebei Finance University, Baoding 071000, China; yunshengshi@mail.bnu.edu.cn

**Keywords:** digital leadership, AI service awareness, AI crafting, proactive service performance, public service motivation

## Abstract

With the development of digital technology and artificial intelligence (AI), numerous studies have focused on the applications and impacts of digital technology in the public sector. However, few studies have explored how frontline public service employees, the core subject of public organizations, can improve their proactive service performance. Based on the model of proactive motivation, this paper investigates the influence of digital leadership on employees’ proactive service performance from a micro perspective, as well as the internal mechanisms and boundary conditions underlying this process. Through an analysis of three-wave questionnaire survey data from 234 employees, this study finds that digital leadership has a positive impact on public employees’ proactive service performance through the serial mediation effects of AI service awareness and AI crafting. Furthermore, as an important boundary condition, employees’ public service motivation strengthens the serial indirect effect of digital leadership on proactive service performance. This paper not only extends the literature on digital leadership by adopting a micro-level perspective within the context of public sector digital transformation but also identifies the individual and contextual antecedents of proactive service performance by examining the interactive effect of public service motivation and leadership. Furthermore, this paper offers valuable implications for the practice of digital transformation in public organizations.

## 1. Introduction

With the prevalence of New Public Management (NPM) reform ideas emphasizing efficiency and outcome orientation in the public sector over the past decades (Lapuente & Van de Walle, 2020), coupled with the rapid growth of artificial intelligence (AI) technologies in recent years, global public sector governance is undergoing a profound paradigm shift from “digitalization” to “intelligentization” to make customer-centric, outward-oriented public services faster and more effective (Ke et al., 2026). As the direct providers of public services, frontline public employees’ service delivery directly affects the quality of public services and the realization of public interests (Eldor, 2018; Plesner et al., 2018). Frontline public employees work directly with citizens. The quality and proactivity of their service behaviors directly affect whether they can quickly adjust their service models and respond to citizens’ service needs, and further influence whether policy objectives can be translated into actual public value (Pawlowski & Scholta, 2023). Given the notable advantages of AI technologies in data collection, analysis, and prediction, how to effectively leverage AI to enhance public employees’ proactive service performance has become a key concern for both public administration researchers and practitioners (Ke et al., 2026).

Proactive service performance refers to employees’ self-initiated, forward-looking, and persistent service behavior, representing a typical manifestation of service-oriented proactive behavior (Rank et al., 2007). With the development of digital and AI technologies, the public service environment is undergoing a significant transformation. Citizens’ needs and expectations for public services are constantly changing. For frontline public employees, achieving traditional job performance is no longer sufficient to meet citizens’ diverse, growing, and rapidly changing service demands (Zhan et al., 2024). For this reason, as a customer-centric, long-term-oriented proactive behavior, proactive service performance goes beyond one’s job description and is not mandated by the organization (Peng et al., 2023; Sun et al., 2023). Proactively anticipating, meeting, and responding to citizens’ service needs helps public employees address service challenges from citizens (Rank et al., 2007; Raub & Liao, 2012). In the context of AI application in the public sector, public employees with high levels of proactive service performance can effectively utilize AI technologies to maintain close connections with citizens, actively engage in public service behaviors, and quickly seek feedback while responding to citizens’ needs (Hur & Shin, 2024). In particular, AI-supported public service delivery requires employees not only to perform traditional service tasks but also to proactively adapt to digitally mediated workflows and AI-enabled service interactions. With the help of AI tools, public employees who exhibit high levels of proactive service performance can also anticipate citizens’ service needs and provide extra public services (Yan et al., 2023). Through continuous spontaneous proactive service behaviors, such conduct is of great value for improving public satisfaction with public services and fostering positive government–citizen relationships (Ke et al., 2026), and is directly instrumental to the success of organizational digital transformation (Busch et al., 2018). Because of these important positive effects, proactive service performance has become a focus of research and practice in multiple industries, including the public sector. Therefore, understanding how frontline public employees translate AI-enabled changes into proactive service behaviors is theoretically and practically important.

Against this background, emerging literature has begun to examine the potential antecedents of frontline public employees’ proactive service performance, such as emotional labor and cultural intelligence at the individual level (Al Shaer et al., 2023), as well as inclusive climate and customer support at the contextual level (Ke et al., 2026; H. Zhang et al., 2021). Although these studies offer valuable insights, they generally overlook how leadership may shape frontline employees’ proactive service performance in the context of AI applications. This is a notable gap, as positive leadership factors are widely recognized as important drivers of employees’ positive psychology, attitudes, and behaviors in the service process, which can enhance service performance and ultimately improve organizational outcomes (Schwarz et al., 2020; Thaler & Helmig, 2016). In this vein, we propose that, in the context of AI entering public organizations, digital leadership, which refers to a leader’s ability to leverage digital technologies to guide organizations through digital transformation and achieve organizational objectives (Cheng et al., 2025), may be a key leadership style driving public employees’ proactive service performance. Digital leadership is generally regarded as a critical driver for improving the level of digital transformation, enhancing governance effectiveness, maintaining public value, and increasing citizen satisfaction in public organizations (Ushaka Adie et al., 2024). We argue that digital leadership can guide employees to engage in digital activities and provide incentives that encourage them to participate more proactively in public services using AI technologies (Q. Lin, 2024), ultimately enhancing their proactive service performance. However, despite these potential positive effects of digital leadership, extant literature has predominantly focused on its macro-level impact on technology adoption or policy formulation, and has yet to sufficiently explain how digital leadership shapes public employees’ service psychology and proactive behavior at the micro level (Ushaka Adie et al., 2024). This omission limits our understanding of how digital leadership facilitates frontline employees’ proactive adaptation to AI-enabled public service environments through AI-related cognition, proactive work redesign, and service behaviors. Therefore, to advance scholarship on public employees’ proactive service performance and digital leadership, this paper examines how digital leadership promotes public employees’ proactive adaptation to AI-enabled public service environments, as well as the underlying mechanisms and boundary conditions of this process.

The model of proactive motivation provides a robust theoretical framework for exploring how public employees proactively adapt to AI-enabled public service environments under digital leadership. This model emphasizes the process by which individuals’ proactive work behaviors are generated, specifically that positive factors from the work context (e.g., a positive leadership style) can foster individuals’ proactive motivational states and drive proactive work behaviors, ultimately yielding positive work outcomes (Parker et al., 2010). In the highly standardized and risk-averse context of public sector digitalization, frontline employees’ application of digital technologies requires not only continuous personal learning but also vision-sharing and psychological empowerment from leaders to ignite proactive states (Ertiö et al., 2024). In detail, as a positive leadership contextual feature, digital leadership can help employees better understand the relevance and applicability of AI in public service work (Q. Lin, 2024). AI service awareness refers to employees’ awareness, understanding, or knowledge of AI use in public services (Flavián et al., 2022). In the present study, we regard this construct as a cognitive basis within the proactive process. By encouraging employees to acquire digital knowledge, creating AI-related learning opportunities, and communicating a clear vision for digital public services, digital leadership can strengthen employees’ awareness of how AI may support service improvement. On this cognitive basis, employees are more likely to recognize how AI can be meaningfully incorporated into their work, which may subsequently support AI crafting as an AI-focused form of proactive work redesign (W. Li et al., 2024; F. Xu et al., 2026). By proactively redesigning their work methods and processes around AI technologies, public employees can better navigate AI-enabled changes in public service delivery and collaborate with AI in service work, ultimately contributing to proactive service performance (Dong et al., 2025; Y. Xu et al., 2026).

Furthermore, the model of proactive motivation posits that, beyond contextual factors from leadership, employees’ positive personal traits can interact with these contextual factors to jointly enhance proactive work states and behaviors, bringing about even more positive work outcomes (Parker et al., 2010). In other words, the positive effects of digital leadership vary for individuals with different characteristics. Specifically, we argue that public employees’ public service motivation, defined as “an individual’s orientation to delivering services to people with a purpose to do good for others and society” (Perry & Hondeghem, 2008, p. 7), serves as a critical moderator. We argue that PSM serves as a key moderator of employees’ proactivity in public organizations. Employees with high PSM have stronger motivation and intrinsic drive to serve the public (Ritz et al., 2016) and are more willing to learn AI-related digital technologies and work methods in order to improve public service (C.-A. Chen & Hsieh, 2015). In our model, however, PSM is not treated as a direct antecedent of AI crafting. Rather, employees with high PSM are more likely to interpret digital leaders’ AI-related guidance through the lens of public value, which strengthens the translation of digital leadership into AI service awareness and further supports the subsequent AI-focused work-redesign process. Therefore, we argue that public service motivation interacts with digital leadership to strengthen the positive serial mediated relationship between digital leadership and employees’ proactive service performance.

**Figure 1:**
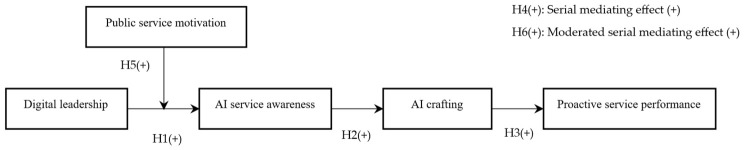
Conceptual Model.

In summary, we construct a moderated sequential mediation model (as shown in Figure 1) to examine the impact of digital leadership on public employees’ proactive service performance, with the mediating roles of AI service awareness and AI crafting, and the boundary condition of PSM. We expect to make the following four contributions to the existing literature. First, it extends research on proactive service performance by explaining how public employees proactively adapt to AI-enabled public service environments during digital transformation. Second, it enriches digital leadership research in the public sector by moving beyond conventional leadership–behavior models and uncovering how digital leadership facilitates employees’ AI-related cognitive and work-redesign processes. Third, it advances understanding of AI crafting by conceptualizing it as an AI-focused form of proactive work redesign through which employees proactively reshape tasks, relationships, and work cognition in response to AI-enabled changes in public service delivery. Finally, it expands the application of public service motivation in digital contexts by highlighting how employees’ intrinsic prosocial motivation strengthens the link between digital leadership and AI service awareness, thereby supporting subsequent AI-focused work redesign.

## 2. Theory and Hypotheses

### 2.1. Proactive Service Performance

For the past two decades, scholars in the service field have consistently regarded frontline employees’ proactive behavior as an important research topic, because the service performance of frontline employees is critical to organizational performance and service success (Tajeddini et al., 2020). As a widely discussed concept of proactive behavior, proactive service performance has attracted the attention of scholars in service sectors and organizational research, defined as an “individual’s self-started, long-term oriented, and persistent service behavior that goes beyond explicitly prescribed performance requirements” (Rank et al., 2007, p. 366). Therefore, proactive service performance is widely regarded as a proactive behavior in which service employees use their own resources and act on their own willingness to provide future services to customers, with the ultimate goal of providing diversified services to customers (Raub & Liao, 2012). Proactive service performance has been extensively confirmed by scholars to provide customers with long-term oriented service, to meet customers’ changing needs, and to play a positive role in enhancing customer satisfaction (M. Chen et al., 2017; Raub & Liao, 2012; Yan et al., 2023).

Given these positive effects, recent public administration scholars have applied this concept to frontline public employees (Ke et al., 2026). Delivering high-quality public services has always been an important concern for public management scholars and practitioners (Lapuente & Van de Walle, 2020; Ramseook-Munhurrun et al., 2010). The proactive service performance of public employees can enable them to deliver services beyond citizens’ expectations and meet their needs and demands, which effectively improves government service levels and efficiency, ultimately leading to increased citizen satisfaction as well as positive government trust and citizen–government relationships (Ke et al., 2026). Despite these positive effects, research on frontline public employees’ proactive service performance remains rather limited, even though meta-analytic evidence from service and business sectors has confirmed that leadership in organizations and employees’ individual characteristics are important predictors of employees’ proactive service performance (Yan et al., 2023, 2025). Given this consideration, integrating leadership and individual characteristics to explore antecedents of frontline public employees’ proactive service performance has urgent theoretical value.

Having established the relevance of proactive service performance, it is worth noting that in this study, although both proactive service performance and AI crafting originate from employees’ self-initiated agency and are recognized as forms of proactive behavior (Ke et al., 2026; Rank et al., 2007; W. Li et al., 2024), they differ fundamentally in multiple aspects. In terms of behavioral target, proactive service performance is directed externally towards customers or citizens, aiming to anticipate and exceed their service expectations (Ke et al., 2026), whereas AI crafting is internally focused on employees’ own work, reflecting a bottom-up approach in which individuals make changes to their work design and working environment in response to AI integration (W. Li et al., 2024; Du et al., 2026). Regarding the domain of behavior, proactive service performance “specifically captures proactive behavior in the service domain” (Rank et al., 2007, p. 364); in contrast, AI crafting is viewed as a “domain-specific kind of job crafting involving AI” (W. Li et al., 2024, p. 2), focusing on how employees proactively and spontaneously adjust their daily work in the context of AI (Laukkarinen, 2025). In terms of the purpose of behavior, proactive service performance aims to enhance citizen satisfaction and public service outcomes (Ke et al., 2026; Rank et al., 2007), representing a typical outcome-oriented proactive behavior construct ultimately intended to improve citizens’ satisfaction with public services (Yan et al., 2025). By comparison, AI crafting concerns employees’ proactive redesign of tasks, relationships, and work cognition around AI, thereby improving their acceptance of and engagement with AI-enabled work (W. Li et al., 2024).

In short, AI crafting and proactive service performance differ in target, domain, and theoretical role. AI crafting is internally oriented and work-design focused: it concerns how employees proactively redesign their own tasks, relationships, and work cognition around AI. Proactive service performance is externally oriented and service-outcome focused: it concerns how employees anticipate, respond to, and exceed citizens’ service needs. Thus, AI crafting is positioned as a mediating work-redesign process, whereas proactive service performance is the downstream service-oriented outcome. Prior research also suggests that AI crafting can facilitate service-domain proactive behaviors, such as service innovation, by helping employees reshape their roles around AI (Y. Xu et al., 2026). Accordingly, AI crafting holds significant potential to predict employees’ proactive service performance in the public service domain.

### 2.2. Digital Leadership

The continuous advancement of digital technologies is driving organizations to transform in response to the demands of digital transformation (Q. Lin, 2024; Philip et al., 2023). A critical factor in this process is whether leaders can effectively guide digital strategies and shape innovation trajectories (Yao et al., 2024), thereby fostering digital leadership (Cheng et al., 2025). Digital leadership arises from the deep integration of traditional leadership approaches with digital competencies in the digital era. It emphasizes the use of digital thinking to steer organizations toward successful digital transformation (Gao & Gao, 2024; Sawy et al., 2016). Unlike traditional leadership styles, such as transformational leadership, digital leadership retains essential elements, including motivating followers and aligning values, while incorporating digital knowledge, experience, and mindset. Moreover, it places greater emphasis on the development of digital strategies, technological capabilities, and a digitally oriented organizational culture (AlNuaimi et al., 2022; Gao & Gao, 2024; Magesa & Jonathan, 2022). These distinctive characteristics enable digital leadership to play a crucial role in facilitating digital transformation. Digital leaders can directly enhance organizational performance through the effective use of communication channels (Nieken, 2023; Öngel et al., 2024) while also fostering an innovation-supportive environment by promoting knowledge sharing (Latif et al., 2025). Furthermore, digital leadership not only drives business transformation and increases the complexity of talent management (Fernandez-Vidal et al., 2022) but also contributes positively to the development of digital organizational culture, the strengthening of digital capabilities, and the achievement of organizational sustainability (Cheng et al., 2025).

Meanwhile, the application of digital leadership has expanded beyond the corporate domain to the public sector, where it has become an urgent necessity for enhancing governance effectiveness. In the public sector, digital leadership is viewed as an approach that guides and gives meaning to government digital transformation, while ensuring the creation of public value and the lawful use of technology (Branderhorst & Ruijer, 2024). It requires leaders to possess a distinctive combination of capabilities, skills, knowledge, and experience, and to develop strategies aligned with public service objectives (Ushaka Adie et al., 2022). The importance of digital leadership has been increasingly recognized (e.g., Khan et al., 2024; Namatovu & Kyambade, 2025). Scholarly attention to this topic has grown substantially in recent years (e.g., Branderhorst & Ruijer, 2024; Khan et al., 2024, 2025; Namatovu & Kyambade, 2025; van Roekel et al., 2025). Existing studies primarily focus on the development of competency frameworks for government leaders and rely heavily on qualitative methodologies, while paying insufficient attention to the exploration of specific micro-level mechanisms (Branderhorst & Ruijer, 2024; van Roekel et al., 2025). In fact, uncovering how digital leadership influences individuals at the micro level is not only essential for translating digital transformation from high-level strategic design into effective implementation, but also critical for improving public service quality and enhancing governmental efficiency (Namatovu & Kyambade, 2025). Accordingly, recent research has called for more in-depth investigation into the mechanisms through which leaders shape employee behavior in digital contexts (Nielsen et al., 2024).

In conclusion, this study adopts the model of proactive motivation proposed by Parker et al. (2010) as its theoretical foundation to examine how digital leadership influences proactive service performance among public employees. The model identifies leadership style as a key contextual factor in motivating employees’ proactive behaviors, and it explicates the underlying mechanisms by which leadership shapes employees’ proactive motivational states, drives proactive behaviors, and ultimately influences work outcomes. Moreover, the model of proactive motivation highlights how individual differences interact with situational factors to jointly influence the motivational processes underlying proactive behavior. This perspective enables a comprehensive examination of the synergistic effects between leadership and individual characteristics among public sector employees. Such an approach aligns closely with the conceptual underpinnings of digital leadership (Branderhorst & Ruijer, 2024; Tigre et al., 2023), thereby providing strong theoretical support for the present study.

### 2.3. The Model of Proactive Motivation

As AI increasingly becomes embedded in the public service process, such as delivering public services and participating in public policy decision-making, more attention has been paid to the process by which public employees proactively provide services (Hjaltalin & Sigurdarson, 2024; Neumann et al., 2024). Employees’ positive states and proactive behaviors are of great value for public organizations to improve the quality of public services and gain citizens’ trust (Luu, 2018; Parker et al., 2006). The model of proactive motivation proposed by Sharon K. Parker and her colleagues provides a theoretical foundation for understanding the process by which employees in organizations proactively seek positive outcomes. This theory argues that employees’ proactive behavior is a goal-driven psychological process that includes goal generation and goal striving (Parker et al., 2010). It also identifies three proximal motivational states: (a) “can do”, (b) “reason to”, and (c) “energized to” (Parker et al., 2010). These three types of motivation enable employees to spontaneously take a future-oriented approach and drive change. The theory indicates that contextual factors and individual differences indirectly influence proactive behaviors and their outcomes by affecting these motivational states (Hirschi et al., 2013; Ng et al., 2021; Parker et al., 2010; A. J. Xu et al., 2022). As an important contextual factor in organizations, leadership significantly influences employees’ proactive processes. For example, transformational leadership can indirectly affect employees’ voice behavior through positive motivational states (G. Zhang & Inness, 2019), while servant leadership encourages employees to take charge at work through prosocial motivation.

In the context of digital intelligence, digital leadership, as a leadership style that actively promotes digital transformation, can positively influence employees’ proactive behaviors and enhance positive outcomes (Alam et al., 2025; G. Zhang & Zhao, 2026). However, existing literature has insufficiently explored this proactive process within public organizations. The model of proactive motivation proposed by Parker et al. (2010) can provide a theoretical basis for this process (Guo et al., 2026; Shi et al., 2025) and offer practical guidance for how public organizations can promote employees’ proactive behaviors and continuously improve the quality of public services (Borah et al., 2022; G. Zhang & Zhao, 2026).

Specifically, we expect that digital leadership, as a positive leadership contextual characteristic, can effectively enhance employees’ AI service awareness (Q. Lin, 2024; Flavián et al., 2022). On this cognitive basis, employees are more likely to recognize how AI can be meaningfully incorporated into public service work (Dong et al., 2025). Such awareness may subsequently support AI crafting, an AI-focused form of proactive work redesign through which employees reshape tasks, relationships, and work cognition in response to AI-enabled changes. We argue that PSM serves as a key moderator of employees’ proactivity in public organizations. Employees with high PSM have stronger motivation and intrinsic drive to serve the public (Ritz et al., 2016) and are able to proactively learn and adapt to AI-related digital technologies, thereby strengthening their AI service awareness and engaging more actively in AI crafting, which in turn positively affects proactive service performance.

### 2.4. Digital Leadership and AI Service Awareness

The model of proactive motivation emphasizes that contextual factors can shape the process through which employees identify proactive opportunities and translate them into future-oriented action (Parker et al., 2010). In the present context, we argue that digital leadership, as a key strategic management capability in the digital age, lies in its ability to enhance employees’ digital technology application capabilities and deepen their understanding of the relevance and applicability of AI in public service work, thereby strengthening their AI service awareness.

First, digital leadership can enhance employees’ self-efficacy in digital technology through digital skills training, which in turn elevates their AI service awareness. In public organizations, due to strict accountability systems and an inherent risk-averse tendency, employees tend to adopt a prudent attitude toward the application of new technologies (Gil-Garcia et al., 2014). Digital leadership can address this dilemma by systematically improving employees’ capabilities (AlNuaimi et al., 2022). Specifically, digital leaders are not only formulators of strategic visions but also enablers of technical capabilities (Shin et al., 2023). They transform abstract AI technology into concrete, perceivable, learnable, and operable skills for employees by designing practical training, establishing demonstration projects, and building learning organizations (Androutsopoulou et al., 2024). This process significantly lowers the technical threshold for AI applications, thus effectively enhancing individuals’ self-efficacy in using AI technology (Gil-Garcia et al., 2014). When employees are confident in their ability to master AI tools to complete work tasks, their resistance to exploring and accepting AI technology is greatly reduced, and they develop the awareness to apply AI in public services (Imran et al., 2025).

Second, digital leadership can strengthen employees’ AI service awareness by reinforcing their recognition of public service values and the appropriate, ethical use of AI. AI deployment in the public sector raises ethical challenges related to algorithmic fairness, data privacy, transparency, and accountability (van Roekel et al., 2025). These challenges are directly tied to employees’ AI service awareness, as AI-supported public services must meet expectations of legality, fairness, transparency, and accountability. Employees’ awareness thus depends not only on technical understanding, but also on whether they perceive AI use as responsible and compatible with public service values. Prior research confirms that while AI creates service opportunities, it also raises concerns about transparency, accountability, and ethical use (Wirtz et al., 2019). Similarly, digital leadership in the public sector should be linked to public value rather than technology alone (van Roekel et al., 2025). From this ethical perspective, digital leadership helps employees interpret AI as a service-enabling resource to be used within the boundaries of public values and professional judgment, reducing ambiguity about AI use. By communicating AI usage norms and emphasizing accountability in AI-assisted service work, digital leadership strengthens employees’ understanding of when, why, and how AI can support public service delivery without violating public-sector requirements (Wang et al., 2025). This clarification of application boundaries further reduces employees’ concerns about potential errors in AI use (Wu et al., 2024), thereby reinforcing AI as a meaningful and legitimate tool for public service delivery.

Finally, digital leadership can reduce employees’ uncertainty and perceived threat surrounding AI use through supportive measures, thereby strengthening their AI service awareness. First, digital leadership can create a supportive atmosphere for employees and arouse their intrinsic energy through organizational support and environmental restructuring (Zulu & Khosrowshahi, 2021). Second, by securing budget funds for AI technology application and providing digital technical support, digital leadership lays a solid resource foundation for employees’ AI application (Tigre et al., 2023). Third, digital leadership is committed to the redesign of work processes, positioning AI as an auxiliary tool to enhance human decision-making and service capabilities rather than a substitute for employees, thus strengthening employees’ sense of subjectivity and control (Erhan et al., 2022). When organizations provide a clear strategic direction, sufficient resource support, and a supportive technical application environment, employees may experience less threat and ambiguity surrounding AI use, which helps them recognize AI as a legitimate and meaningful resource for public service work (Namatovu & Kyambade, 2025). In summary, we propose the following hypothesis:

**Hypothesis** **1.** *Digital leadership has a positive influence on employees’ AI service awareness*.

### 2.5. The Mediating Roles of AI Service Awareness and AI Crafting

Based on the model of proactive motivation, employees’ cognitive interpretation of their work context can support proactive goal generation and subsequent proactive action (Parker et al., 2010). In the digital public service context, digital leadership can enhance employees’ AI service awareness by helping them understand how AI may support public service work (Philip et al., 2023). Once employees develop such awareness, they are more likely to consider how AI can be incorporated into their daily tasks. In this process, AI crafting functions as an AI-focused proactive work-redesign mechanism. It has been conceptualized as a work-redesign process through which employees reshape their tasks, relationships, and work cognition in response to AI-related work changes (W. Li et al., 2024). It is defined as “volitional actions to shape, mold, and redefine one’s job in response to AI” (W. Li et al., 2024, p. 2), and refers to employees’ proactive adjustment of work tasks, relationships, and cognition to integrate AI tools and improve human–AI collaboration (Dong et al., 2025; W. Li et al., 2024; Y. Li et al., 2025).

First, AI service awareness can facilitate employees’ engagement in AI crafting because it provides a cognitive basis for employees to understand what AI can do, why AI is relevant to service work, and what capabilities are needed to use AI effectively. From the perspective of the model of proactive motivation, such awareness provides a cognitive basis for proactive goal generation by helping employees identify AI-related opportunities for service improvement and recognize how AI may be meaningfully incorporated into their work. Employees with stronger AI service awareness are more likely to see AI as relevant to their service tasks, understand where AI can be applied, and identify the skills needed for meaningful AI use.

On the one hand, employees with AI service awareness recognize that a higher level of AI application can support better public service delivery (Huang & Gursoy, 2024). This recognition may encourage them to learn AI technologies, improve their AI application capabilities, and optimize work processes for human–AI collaboration (Tims et al., 2013). In other words, AI service awareness helps employees connect the value of AI with the skills required to use AI in practice. When employees understand both the potential of AI and the capabilities needed for its application, they are more likely to move beyond passive acceptance of digital tools and actively explore how AI can be embedded in their work. On the other hand, AI service awareness may also make employees more likely to experiment with AI in public service work. When employees recognize the advantages of AI for public service delivery, they are more likely to identify concrete opportunities for using AI to improve tasks, collaboration, and service processes (Wilson & Daugherty, 2018). Based on their own agency, employees can then explore how to interact with AI, collaborate with AI more effectively, and redesign work through AI crafting (Larson & DeChurch, 2020). Therefore, AI service awareness helps employees reinterpret their work goals in the context of digital and intelligent technologies. By developing and applying AI-related skills, employees can use AI’s analytical capabilities to deliver higher-quality public services and better accomplish their work (Dióssy et al., 2025).

This theorized ordering is also consistent with recent AI-related organizational research. Prior studies suggest that employees’ cognitive recognition or appraisal of AI-related work changes can precede crafting responses. For example, He et al. (2024) found that employees’ challenge appraisals toward AI are associated with job crafting, which subsequently contributes to service performance. Mo et al. (2024) showed that AI awareness can encourage employees to engage in different forms of job crafting. F. Xu et al. (2026) further examined how AI awareness influences promotion-focused and prevention-focused job crafting. Although these studies do not examine AI service awareness in public organizations specifically, they provide convergent support for the broader argument that employees’ awareness or appraisal of AI-related changes can serve as an antecedent to proactive work redesign.

**Hypothesis** **2.** *AI service awareness has a positive influence on employees’ AI crafting*.

Next, we argue that AI crafting can further improve employees’ proactive service performance. If AI service awareness provides the cognitive basis for approaching AI-enabled service work, AI crafting represents the AI-focused work-redesign process through which employees translate such awareness into concrete changes in tasks, relationships, and work cognition. Through AI crafting, employees can reposition their tasks, cognition, and relationships with AI, thereby improving their ability to provide proactive public services. Previous research has shown that AI crafting can promote proactive service behavior by helping employees proactively redesign their work in response to AI-enabled changes (Y. Xu et al., 2026), which provides theoretical evidence for understanding the relationship between AI crafting and proactive service performance.

First, AI crafting enables employees to reallocate work resources and redesign task arrangements more effectively. Public service work often involves repetitive administrative procedures, information processing, and routine communication. By using AI to support standardized tasks such as repetitive administrative approval and data entry, employees can devote more attention to complex issues, interaction with service recipients, and risk prevention (Acquah, 2025). Employees can also use AI’s ability to process and analyze large-scale data to compensate for their cognitive limitations. This may help them move from passively responding to citizens’ expectations to proactively predicting service demands and providing more future-oriented public services (Wrzesniewski & Dutton, 2001).

Second, AI crafting can strengthen employees’ sense of autonomy and competence in AI-enabled work (W. Li et al., 2024). When AI helps handle routine tasks, employees’ cognitive resources can be released, allowing them to focus on work that requires judgment, decision-making, and creativity (Rozkwitalska et al., 2022). At the same time, AI crafting may encourage employees to understand, verify, and use explainable AI technologies in public service work, thereby strengthening their confidence and perceived capability (Topçuoğlu et al., 2023). AI can also assist employees in learning, help them develop new skills, and enhance their sense of accomplishment by providing personalized guidance (Karny et al., 2024). These changes make employees more capable of responding actively to service problems and improving service quality.

Third, AI crafting can reshape employees’ relationships with AI and promote more effective human–AI collaboration. Employees who engage in AI crafting are less likely to regard AI only as a technical system or potential competitor. Instead, they may treat AI as a work partner and increase the level of AI participation in their work (He et al., 2025). When employees form sufficient cognitive and value recognition of AI technology, they are more likely to explore new models of human–AI collaboration, redesign work processes to maximize AI effectiveness, and improve proactive service performance (Wilson & Daugherty, 2018; C.-P. Lin & Chen, 2025).

Taken together, AI crafting helps employees transform their awareness of AI into proactive service performance by redesigning work tasks, strengthening competence, and developing more effective human–AI collaboration. Thus, we propose the following hypothesis:

**Hypothesis** **3.** *AI crafting has a positive influence on employees’ proactive service performance*.

Building on the above arguments and the model of proactive motivation, we further propose that AI service awareness and AI crafting serially mediate the relationship between digital leadership and employees’ proactive service performance. Parker et al. (2010) conceptualize proactivity as a goal-directed process that involves proactive goal generation and proactive goal striving. In the present context, AI service awareness supports proactive goal generation by helping employees identify AI-related opportunities for improving public service work and understand the relevance of AI to their service tasks (Vial, 2019). AI crafting reflects the subsequent work-redesign process through which employees act on such recognition and proactively redesign tasks, relationships, and work cognition around AI (Lai & Rau, 2024). Accordingly, digital leadership is expected to be associated with proactive service performance through a sequential cognitive and work-redesign pathway in which digital leadership strengthens AI service awareness, AI service awareness facilitates AI crafting, and AI crafting ultimately contributes to proactive service performance. Therefore, we propose the following hypothesis:

**Hypothesis** **4.** *AI service awareness and AI crafting serially mediate the positive relationship between digital leadership and employees’ proactive service performance*.

### 2.6. The Moderating Role of Public Service Motivation

The model of proactive motivation indicates that the impact of contextual factors, such as leadership style, on individuals’ proactive states can be shaped by individual characteristics (Parker et al., 2010). Individual characteristics influence whether and how employees interpret contextual cues as meaningful for proactive action. Public service motivation (PSM) refers to an individual’s intrinsic drive to serve the public (Perry & Wise, 1990). Public employees with high PSM derive their core work motivation from an intrinsic commitment to serving the public interest (Ritz et al., 2016). In the context of public-sector digital transformation, this value orientation is especially relevant because employees need to interpret whether AI-related changes are merely technical requirements or meaningful tools for improving public service.

We argue that PSM strengthens the relationship between digital leadership and AI service awareness because it creates a value-based fit between leaders’ digital guidance and employees’ public service orientation. Digital leadership not only provides technical support and digital resources, but it also communicates the public value and strategic relevance of AI applications in service work. Employees with high PSM are more likely to interpret such leadership signals through their commitment to serving citizens and improving public outcomes. They tend to understand AI technology as a means to achieve public service goals and improve governance efficiency (Wright et al., 2013). As a result, digital leadership is more likely to be translated into AI service awareness among employees with high PSM because these employees can connect AI use with their own public service values.

PSM also shapes how employees interpret the support and resources provided by digital leadership. Digital leadership may build an environment conducive to AI application by providing training, resources, and support. Employees with high PSM are more likely to view these measures as enabling conditions for fulfilling their public service duties rather than as additional technical burdens (Ritz et al., 2016). In this sense, AI-related learning and application are not separated from their work identity. Instead, they may be regarded as an extension of their responsibility to improve public service. Therefore, these employees are more inclined to use the opportunities created by digital leadership to improve their capabilities and to understand the service relevance of AI (Holt & Piatak, 2023), which facilitates the formation of AI service awareness.

By contrast, employees with low PSM may rely more on external rules or routine job requirements when interpreting organizational changes (Christensen et al., 2017). For them, AI-related changes promoted by digital leadership may be perceived less as opportunities to enhance public service and more as additional work demands or adjustment costs (Masukela et al., 2023). Consequently, the same digital leadership practices may be less effective in helping them form AI service awareness. In summary, PSM does not simply make employees more motivated in a general sense. Rather, it specifically strengthens the link between digital leadership and AI service awareness by aligning leaders’ digital guidance with employees’ public service values. Thus, we propose the following hypothesis:

**Hypothesis** **5.** *Public service motivation moderates the positive relationship between digital leadership and AI service awareness, such that this positive relationship is stronger when public service motivation is high*.

### 2.7. An Integrated Model

Based on the above hypotheses, we further propose an integrated moderated serial mediation model. Digital leadership may first enhance employees’ AI service awareness by helping them understand the service value, work relevance, and appropriate use of AI. AI service awareness then supports employees’ engagement in AI crafting, through which they redesign tasks, relationships, and work cognition to integrate AI into public service work. This process ultimately contributes to proactive service performance.

The strength of this serial process is likely to depend on employees’ PSM. When employees have high PSM, they are more likely to interpret AI-related guidance from digital leaders through the lens of public value. For these employees, AI use is more likely to be understood as a way to improve service quality and fulfill public responsibilities rather than as an additional technical requirement (Masukela et al., 2023). This stronger AI service awareness may further support their engagement in AI crafting by helping them connect AI use with public service responsibilities (Ritz et al., 2016; Holt & Piatak, 2023), ultimately improving proactive service performance. In contrast, for employees with low PSM, the effect of digital leadership may be weaker at the first stage because AI-related guidance is less likely to be connected with their public service values (Christensen et al., 2017). Therefore, the indirect impact of digital leadership on proactive service performance through AI service awareness and AI crafting is expected to become stronger as employees’ PSM increases (Holt & Piatak, 2023).

In summary, PSM not only moderates the relationship between digital leadership and AI service awareness but also shapes the strength of the entire serial indirect pathway from digital leadership to proactive service performance. Thus, we propose the following hypothesis:

**Hypothesis** **6.** *Public service motivation moderates the serial indirect effect of digital leadership on employees’ proactive service performance via AI service awareness and AI crafting, such that the indirect effect is stronger when public service motivation is high*.

## 3. Methods

### 3.1. Procedure and Participants

The research subjects are frontline public servants from several sub-districts in City B, China. Their daily administrative work involves tasks such as document processing, citizen request classification, resource allocation, and preliminary decision support, all of which are increasingly assisted by digital technologies, including AI-based systems. Specifically, all respondents regularly use a standardized AI-assisted administrative platform deployed by the local government, which includes functions such as automated document sorting, intelligent case assignment, and predictive analytics for service demand. The platform is uniformly applied across sub-districts, ensuring that all participants have similar exposure to and experience with AI tools in their routine work.

This study was approved by the Institutional Review Boards of the School of Government, Beijing Normal University. With the help of the human resource management departments of the sub-districts and with organizational assistance, we recruited 286 participants to participate in this survey, and a three-wave questionnaire survey was conducted. Before we conducted the survey, all participants provided written informed consent after being fully informed about the study purpose, the voluntary participation, the right to withdraw at any time without penalty, and the confidentiality of their responses. At Time 1 (T1), we collected participants’ demographic information (including age, gender, education level, organizational tenure, and annual income), PSM, and perceived digital leadership of their immediate supervisors. Two weeks later, at Time 2 (T2), we collected data on participants’ AI service awareness and AI crafting. Two weeks later, at Time 3 (T3), we collected participants’ proactive service performance.

Regarding sample collection: at T1, a total of 286 questionnaires were distributed, and 270 valid questionnaires were retained after review, with an effective response rate of 94.4%; at T2, 270 questionnaires were distributed, and 257 valid samples were retained after review; at T3, 257 questionnaires were distributed, and 234 valid questionnaires were retained after review, resulting in a final effective sample response rate of 81.82%.

The demographic characteristics of the sample are as follows: in terms of gender, 164 were male (70.1%) and 70 were female (29.9%); in terms of age, the largest proportion of the sample was aged 20–30; in terms of education level, the majority held a bachelor’s degree; the average organizational tenure of all participants was 6.50 years; in terms of family annual income, the largest proportion had a family annual income of 100,000 to 200,000 CNY. We have provided the demographic information of participants in Table 1.

### 3.2. Measures

We used validated scales from prior studies and adapted them to the Chinese public-sector context. In the process of translating English scales into Chinese, the study strictly followed the back-translation procedure proposed by Brislin (1970). To ensure that the items fit the research context, we adjusted the wording or instructions according to the Chinese government context. All measurements used a 5-point Likert scale (1 = “strongly disagree”, 5 = “strongly agree”).

Digital leadership (T1): A 6-item scale developed by Lyu (2024) was adopted, with a representative item including “My leader is an expert in digitization”. The Cronbach’s α of this scale in this study was 0.91. The composite reliability (CR) was 0.91, and the average variance extracted (AVE) was 0.64.

Public service motivation (T1): A 5-item scale used by Wright et al. (2013) was adopted, with a representative item including “Meaningful public service is very important to me”. The Cronbach’s α of this scale in this study was 0.84. The CR was 0.85, and the AVE was 0.53.

AI service awareness (T2): The 3-item AI service awareness scale used by Flavián et al. (2022) was adapted, with a representative item including “I was very aware of AI used in my service work”. The Cronbach’s α of this scale in this study was 0.76. The CR was 0.79, and the AVE was 0.56.

AI crafting (T2): A 6-item scale developed by W. Li et al. (2024) was adopted, with a representative item including “When working with AI, I introduce new approaches on my own to improve my work”. The Cronbach’s α of this scale in this study was 0.90. The CR was 0.92, and the AVE was 0.65.

Proactive service performance (T3): A 5-item scale used by Hur and Shin (2024) was used, with a representative item including “I anticipate issues or needs citizens might have and proactively develop solutions”. The Cronbach’s α of this scale in this study was 0.85. The CR was 0.86, and the AVE was 0.56.

Control variables (T1): Previous studies have shown that individual demographic characteristics (e.g., gender, age, education level, organizational tenure, and family annual income) may affect individuals’ proactive work behaviors (Crant, 2000). These factors were included as control variables in this study.

All measurement items are presented in Table A1 in Appendix A.

## 4. Results

### 4.1. Confirmatory Factor Analysis

Confirmatory factor analysis (CFA) was conducted using Mplus 8.0 (Muthén & Muthén, Los Angeles, CA, USA) software. The results showed (as shown in Table 2) that the five-factor model (digital leadership, AI service awareness, AI crafting, proactive service performance, PSM) had the best fit compared with other competing models (χ^2^/*df* = 1.39, CFI = 0.93, TLI = 0.92, RMSEA = 0.04, SRMR = 0.06). Furthermore, we followed the recommendation of Fornell and Larcker (1981) and calculated the square root of the average variance extracted (AVE) for each study variable. These values were then compared with the correlations between the study variables. As shown in Table 3, the square root of the AVE for each variable (presented in parentheses on the diagonal) is greater than its correlations with other variables. In addition, we calculated the Heterotrait-Monotrait Ratio (HTMT) values for all research variables. The results showed that these values ranged from 0.32 to 0.63, all below the threshold of 0.90 (Henseler et al., 2015). The results indicate satisfactory discriminant validity among the study variables.

### 4.2. Testing Common Method Bias

Although this study employed a three-wave time-lagged questionnaire design, common method bias (CMB) might still be a concern. Following Podsakoff et al. (2003), we first conducted Harman’s single-factor test as a statistical remedy. The results revealed five common factors with eigenvalues above 1, collectively explaining 57.64% of the total variance, and the first factor accounted for 27.61% of the variance, which is well under the 40% cutoff. Thus, CMB is unlikely to be a serious issue in this study. As an additional check, we performed confirmatory factor analysis (CFA) to compare several alternative factor structures. A single-factor model, in which all items loaded onto one latent variable, exhibited poor fit (χ^2^/*df* = 2.91, CFI = 0.70, TLI = 0.69, RMSEA = 0.10, SRMR = 0.12), considerably worse than that of our hypothesized model (χ^2^/*df* = 1.39, CFI = 0.93, TLI = 0.92, RMSEA = 0.04, SRMR = 0.06). This comparison suggests that the theoretical model is not substantially contaminated by CMB. Finally, drawing on Williams and Anderson (1994), we introduced an unmeasured latent method factor to account for potential method effects. Specifically, we added a CMB factor onto which all items were loaded, and then compared the model with and without this factor. The inclusion of the CMB factor produced only tiny changes in fit indices (CFI, TLI, RMSEA, and SRMR), ranging from 0.00 to 0.03. This further corroborates that CMB does not significantly affect our results (Podsakoff et al., 2003).

### 4.3. Testing Multicollinearity

To examine potential multicollinearity among the study variables, we calculated the tolerance and variance inflation factor (VIF) values for each variable. As shown in Table 3, the tolerance values for all study variables were greater than 0.20, and the VIF values were less than 5, indicating that there is no serious multicollinearity problem.

### 4.4. Descriptive Statistics and Correlation Analysis

Table 4 reports the means, standard deviations, and correlation coefficients for the research variables in this study. The results showed that digital leadership was significantly positively correlated with AI service awareness (*r* = 0.57, *p* < 0.01), AI crafting (*r* = 0.31, *p* < 0.01), and proactive service performance (*r* = 0.29, *p* < 0.01). AI service awareness was significantly positively correlated with AI crafting (*r* = 0.41, *p* < 0.01) and proactive service performance (*r* = 0.37, *p* < 0.01), which provides preliminary evidence for hypothesis testing.

### 4.5. Hypothesis Testing

Path analysis was performed using Mplus 8.0 software to test the research model. The results in Table 5 show that digital leadership was significantly positively associated with AI service awareness (γ = 0.43, *SE* = 0.04, *p* < 0.001), AI service awareness was significantly positively associated with AI crafting (γ = 0.20, *SE* = 0.04, *p* < 0.001), and AI crafting was also significantly positively associated with proactive service performance (γ = 0.46, *SE* = 0.07, *p* < 0.001). The mediating effect test results indicated that the serial mediating effect of digital leadership on proactive service performance through AI service awareness and AI crafting was significant (effect size = 0.04, 95% CI = [0.011, 0.082]). Therefore, Hypotheses 1, 2, 3, and 4 are supported.

**Figure 2:**
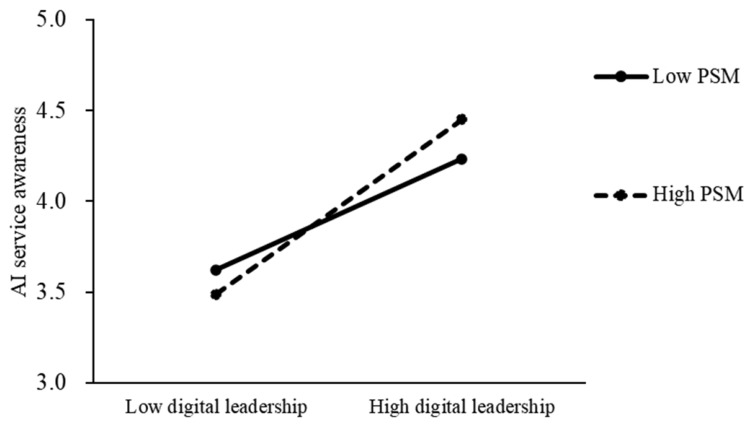
The moderating effect of public service motivation.

Subsequently, the moderating effect of PSM was tested. As shown in Table 4, the interaction term of digital leadership and PSM was significantly positively associated with AI service awareness (γ = 0.16, *SE* = 0.06, *p* < 0.05), indicating that PSM plays a positive moderating role in the relationship between digital leadership and AI service awareness. To clarify the direction of the moderating effect, a moderating effect diagram was drawn through a simple slope test. As shown in Figure 2, compared with low PSM (*b* = 0.34, *p* < 0.001), the positive relationship between digital leadership and AI service awareness was stronger when frontline public servants had a high level of PSM (*b* = 0.53, *p* < 0.001), supporting Hypothesis 5.

In addition, Hypothesis 6 states that PSM moderates the serial mediating effect of digital leadership on proactive service performance through AI service awareness and AI crafting. To test this moderated serial mediation effect, Mplus 8.0 software was used. The results showed that when PSM was high, the serial mediating effect was 0.05 (95% CI = [0.016,0.094]); when PSM was low, the serial mediating effect was 0.03 (95% CI = [0.008, 0.070]). The difference in the serial mediating effects between high and low PSM was also significant (effect size = 0.02, 95% CI = [0.001, 0.040]). Therefore, Hypothesis 6 is also supported.

## 5. Discussion

### 5.1. Research Conclusions

Based on the model of proactive motivation and using a three-wave survey design, this study provides empirical evidence for how digital leadership affects public employees’ proactive service performance in the context of public-sector digital transformation. Specifically, the findings show that digital leadership positively affects public employees’ proactive service performance through the serial mediating roles of AI service awareness and AI crafting. Moreover, this indirect effect is stronger among public employees with higher public service motivation. This study makes important theoretical contributions to the literature on digital leadership, proactive service performance, and public service motivation in the public sector while also offering practical implications for leaders and public employees during the digital transformation of the public sector. Nevertheless, this study has certain theoretical and methodological limitations. Future research could employ different research methods to further examine the causal dynamics of this process across various public organizations, administrative systems, and cultural contexts, and explore potential alternative mediating mechanisms and boundary conditions through which digital leadership influences public employees’ proactive service performance. Next, we discuss the theoretical implications, practical implications, limitations, and future research directions in detail.

### 5.2. Theoretical Implications

Our paper contributes to the proactive service performance, digital leadership, AI crafting, and public service motivation literature in several ways. First, this study makes an important contribution to the emerging literature on public employees’ proactive service performance. With the continuous deepening of customer-oriented values in organizations worldwide (Kennedy et al., 2003), increasing research has focused on the importance of proactive service performance as a form of proactive behavior (Rank et al., 2007; Yan et al., 2025). However, most studies remain limited to examining the driving processes of proactive service performance among frontline employees in the service industry (e.g., Huo et al., 2019; Yan et al., 2023; Zhu et al., 2017). In the context of the public sector, research is still in its infancy. Although scholars have recently begun to pay attention to the factors influencing public employees’ proactive service performance, these studies have generally overlooked the increasingly integrated AI technology context in public organizations and the facilitating role of leaders within it, even though organizational AI adoption and leadership as contextual factors have long been shown to significantly influence employees’ proactive service behavior (Hur & Shin, 2024; Teng et al., 2025; Rank et al., 2007; Ye et al., 2019). By responding to Ke et al.’s (2026, p. 290) call to “examine the moderating effects at the leadership level and individual level”, and by simultaneously focusing on the roles of digital leadership and public service motivation, our study extends existing research on proactive service performance by explaining how public employees proactively adapt to AI-enabled public service environments and translate digital transformation into proactive service behaviors.

Second, we enrich organizational scholarship on digital leadership in the public sector by extending the understanding of how digital leadership shapes employees’ proactive adaptation to AI-enabled public service environments. Although research on digital leadership in the business sector has made some progress (de Araujo et al., 2021; Tigre et al., 2023), studies on digital leadership in the public sector remain very limited. Existing studies have primarily focused on macro-level outcomes such as policy implementation, public value creation, and organizational performance and strategy (e.g., Branderhorst & Ruijer, 2024; Khan et al., 2024; Ushaka Adie et al., 2024; van Roekel et al., 2025) while paying insufficient attention to how digital leadership influences frontline employees’ AI-related cognition and AI-focused proactive work-redesign behaviors during digital transformation. As AI technologies are increasingly integrated into public service delivery, public employees are no longer merely responsible for traditional service tasks; they are also required to proactively adapt to AI-supported workflows and digitally mediated service interactions (Tangi et al., 2025). Against this backdrop, our study centers on how digital leadership facilitates employees’ cognitive and behavioral transformation in the context of AI adoption (Ahn & Chen, 2022). Specifically, by elucidating the mechanism through which digital leadership enhances employees’ AI service awareness, stimulates their AI crafting behaviors, and ultimately improves their proactive service performance, this study provides theoretical and empirical insights into the micro-level pathways via which digital leadership empowers frontline employees to effectively engage in AI-supported public service delivery. More importantly, these findings provide a more contextually specific account of the proactive motivation process in AI-enabled public service work. Parker et al. (2010) conceptualize proactive behavior as a goal-driven process involving proactive goal generation and proactive goal striving. Extending this logic to the public-sector digital transformation context, our model suggests that employees’ awareness of AI usage in public service work can provide a cognitive basis for recognizing service-improvement opportunities, while AI crafting represents the subsequent proactive behavioral response through which employees act on such recognition. In this way, the study clarifies how a cognition-to-action process may unfold when frontline public employees respond proactively to AI-enabled changes in their work.

Third, we enrich the literature on AI crafting, i.e., a specific manifestation of proactive behavior in the digital era, by examining its positive role among public employees and identifying its critical contextual antecedents and underlying psychological mechanisms. AI crafting has recently garnered significant attention as a pivotal work-redesign mechanism for achieving positive digital outcomes (R. Liu et al., 2025; Y. Xu et al., 2026). Despite its documented benefits, research on the drivers of AI crafting has remained largely individual-centric, overlooking the role of leadership in fostering such behaviors, even though it is widely recognized that proactive behavior is often stimulated by the organizational context (Parker et al., 2006). Furthermore, most studies have only examined the way public employees passively accept AI and the potential consequences of this process (Ahn & Chen, 2022), while neglecting how employees proactively redesign their work around AI-enabled changes (W. Li et al., 2024). Different from this research stream, this study conceptualizes AI crafting as an AI-focused form of proactive work redesign through which public employees proactively adjust tasks, workflows, and service interactions in response to AI-enabled changes (Parker & Grote, 2022). By linking AI crafting to proactive service performance, we provide a new perspective for understanding how public employees translate AI-related work redesign into proactive service outcomes in digitally transforming public organizations. This study deepens our understanding of how AI-focused work-redesign processes shape proactive service outcomes in public organizations.

Finally, we expand the application of public service motivation to the digital context and examine its synergistic effect with digital leadership. As AI technologies are gradually integrated into public service delivery, several recent studies have focused on how public employees’ PSM helps shape positive work outcomes (Adinolfi et al., 2026; Neumann & Schott, 2023). However, research on PSM under the contexts of AI has primarily focused on its direct impact on work psychology and behavior (Ritz et al., 2016), often neglecting the interplay between individual PSM and positive contextual factors, despite evidence that PSM is frequently examined as an important moderator in the relationship between leadership and work outcomes (Hameduddin & Engbers, 2022). Drawing on the model of proactive motivation (Parker et al., 2010), which posits that proactive states emerge from the alignment of individual traits and contextual drivers, this study integrates PSM with digital leadership. This integration enriches our understanding of how public employees’ intrinsic prosocial motivation strengthens proactive adaptation to AI-enabled work environments during organizational digital transformation. Furthermore, by identifying PSM as a key boundary condition, we clarify why the positive effects of digital leadership may vary across individuals, responding to the suggestion by Hameduddin and Engbers (2022) that future research should pay attention to differences in public employees’ PSM under different types of leadership styles, thereby offering a more nuanced perspective on how leadership effectiveness in public organizations depends on employees’ willingness to proactively engage in AI-focused work-redesign behaviors.

### 5.3. Practical Implications

The findings offer several practical implications. First, public organizations may benefit from strengthening leaders’ ability to communicate the service value of digital and AI technologies (Zeike et al., 2019). Our results suggest that digital leadership is associated with employees’ AI service awareness. Therefore, rather than only emphasizing technology adoption as an administrative requirement, leaders may need to explain how AI tools can support public service quality, reduce repetitive work, and help employees respond more proactively to service recipients. Second, public organizations may consider providing employees with more concrete AI-related learning opportunities. Since AI service awareness is linked to AI crafting, employees need not only general encouragement but also practical knowledge of how AI can be used in their specific service tasks (Kong et al., 2021). Workshops, peer sharing, demonstration cases, or task-based training may help employees develop a clearer understanding of what AI can and cannot do in public service work. Third, managers should be cautious but supportive in encouraging AI crafting. The findings show that AI crafting is positively related to proactive service performance (Cheng et al., 2025). This does not mean that employees should freely redesign public service procedures without constraints. Rather, within existing administrative rules and accountability requirements, employees may be given appropriate autonomy to explore how AI can assist routine tasks, improve information processing, or support more proactive communication with service recipients. Finally, the moderating role of PSM suggests that digital transformation practices should be connected to public service values. For employees with strong public service motivation, AI-related changes may be more readily accepted when they are framed as tools for improving service fairness, responsiveness, and public value (Christensen et al., 2017). Therefore, public organizations should not present AI merely as a technical upgrade, but as a possible means to better serve citizens.

### 5.4. Limitations and Future Research Directions

Although this study has certain theoretical and practical implications, it still has certain theoretical and methodological limitations that need to be addressed in future research. First, the sample of this study consists of public employees from Chinese government departments, which may limit the generalizability of the findings to other regions, administrative systems, or cultural contexts, although the Chinese context is appropriate for examining AI application in public service work (Y. Li et al., 2025). Research shows that public-sector digital transformation may unfold differently across countries and levels of government, depending on administrative traditions, technological infrastructure, and accountability arrangements. The extent to which organizations and employees adopt AI technologies also varies across different national and cultural contexts (Yigitcanlar et al., 2023). Future research could test whether the proposed model holds across different types of public organizations and institutional contexts.

Second, we only discussed the boundary condition of public service motivation in the relationship between digital leadership and proactive service performance. The model of proactive motivation suggests that various individual difference factors, such as traits, personality, knowledge, and skills, interact with contextual factors (e.g., digital leadership) to influence employees’ proactive behavior and performance outcomes (Parker et al., 2010). Future research could explore how a broader range of individual personal factors interact with digital leadership to produce positive outcomes. For example, employees with a proactive personality and a high level of learning goal orientation are more likely to actively engage in AI crafting, learn digital technologies, or AI skills, thereby enhancing the positive effects of digital leadership (Kong et al., 2024; Matsuo, 2019).

Third, although the hypothesized mediation and moderated mediation effects were statistically significant, the serial indirect effects were relatively small. This is consistent with prior research indicating that serial mediation effects are often attenuated due to the multiplicative nature of indirect paths (e.g., Lanaj et al., 2014; D. Liu et al., 2025; O’Reilly et al., 2016), limiting their comparability with simpler mediation models. This pattern likely reflects the complexity of the sequential mechanism. Accordingly, the indirect effects should be interpreted with caution, as they may underestimate the overall magnitude of the proposed process.

Fourth, although our theoretical model positions AI service awareness before AI crafting, both constructs were measured at Time 2. Therefore, the present design does not allow us to empirically rule out alternative temporal arrangements, including reciprocal or reversed relationships between these two constructs. Our proposed ordering is grounded in the conceptual distinction between awareness of AI use in service work and proactive behavioral redesign around AI, and is also consistent with emerging AI-related studies that position AI awareness or AI appraisal as an antecedent of crafting responses. Nevertheless, future research should directly compare alternative sequence models using cross-lagged panel designs, experience-sampling methods, or repeated longitudinal measurements.

Finally, we employed multi-wave, self-reported questionnaire data to test the proposed research model. Although this approach helps mitigate common method bias to some extent, it still suffers from potential measurement bias, as all variables were rated by the same participants. The use of multi-source data is encouraged in future research. For instance, proactive service performance could be rated by supervisors rather than relying solely on self-reports, thereby reducing concerns about same-source bias and improving the validity of the measurements. In addition, the single-source sample still suffers from demographic limitations. Males account for a large proportion of the sample, and the overall sample is relatively young. This may affect the interpretation of some study variables. For example, younger employees, due to their stronger learning ability and greater AI knowledge compared with older employees (Subramaniam et al., 2025), are more likely to engage in AI crafting. Furthermore, scholars have pointed out that men are less likely than women to be attracted to public service work, which may affect our understanding of public service motivation and proactive service performance (Bright, 2005). Therefore, future research should adopt more stratified sampling and large-sample surveys to ensure the diversity of sample sources, thereby avoiding the demographic limitations inherent in single-source samples.

## Figures and Tables

**Table 1:** Demographic Information of Participants.

Demographics	Number	Percent
Gender		
Male	164	70.1%
Female	70	29.9%
Age		
<30 years	162	69.2%
31–40 years	60	25.6%
41–50 years	12	5.2%
Education		
Vocational college	19	8.1%
Bachelor’s degree	141	60.3%
Master’s degree	65	27.8%
Doctoral degree	9	3.8%
Family annual income (CNY)		
<100,000	7	3.0%
100,000–200,000	92	39.3%
200,000–300,000	71	30.3%
>300,000	64	27.4%

**Table 2:** Results of Confirmatory Factor Analysis.

Model	χ^2^/*df*	CFI	TLI	RMSEA	SRMR
Five-factor model (DL, ASW, AC, PSP, PSM)	1.39	0.93	0.92	0.04	0.06
Four-factor model (1) (DL + ASW, AC, PSP, PSM)	1.90	0.85	0.83	0.06	0.08
Four-factor model (2) (DL, ASW + AC, PSP, PSM)	1.62	0.90	0.88	0.05	0.07
Three-factor model (DL + ASW + AC, PSP, PSM)	2.09	0.83	0.81	0.07	0.08
Two-factor model (DL + ASW + AC + PSM, PSP)	2.47	0.76	0.75	0.09	0.10
One-factor model (DL + ASW + AC + PSP + PSM)	2.91	0.70	0.69	0.10	0.12

Note. *N* = 234; DL = Digital leadership; ASW = AI service awareness; AC = AI crafting; PSP = Proactive service performance; PSM = Public service motivation; “+” indicates that the variables were combined.

**Table 3:** Tolerance and Variance Inflation Factor (VIF) Results.

Variables	Tolerance Value	VIF Score
Digital leadership	0.55	1.82
AI service awareness	0.61	1.66
AI crafting	0.76	1.32
Public service motivation	0.69	1.45

**Table 4:** Descriptive Statistics and Correlations.

Variable	*M*	*SD*	1	2	3	4	5	6	7	8	9	10
1. Gender	1.70	0.46										
2. Age	2.36	0.59	−0.14 *									
3. Education	3.27	0.66	0.05	−0.08								
4. Organizational tenure	6.50	7.56	−0.05	0.36 **	−0.17 *							
5. Family annual income	2.85	0.93	−0.03	0.01	−0.08	0.09						
6. Digital leadership	3.45	0.90	−0.08	−0.06	−0.02	0.04	0.16 *	(0.80)				
7. AI service awareness	3.99	0.68	−0.12	0.00	0.02	−0.05	0.14 *	0.57 **	(0.75)			
8. AI crafting	4.21	0.38	−0.02	−0.08	−0.01	−0.01	0.01	0.31 **	0.41 **	(0.81)		
9. Proactive service performance	4.26	0.51	−0.03	0.05	0.00	0.05	0.05	0.29 **	0.37 **	0.42 **	(0.75)	
10. PSM	3.70	0.62	−0.03	−0.05	−0.07	0.05	0.03	0.50 **	0.30 **	0.38 **	0.29 **	(0.73)

Note. *N* = 234. Gender: female = 1, male = 2; Age: under 20 = 1, 20–30 = 2, 30–40 = 3, 40–50 = 4, 50–60 = 5, above 60 = 6; Education: technical school/high school or below = 1, vocational college = 2, bachelor’s degree = 3, master’s degree = 4, doctoral degree = 5; Family annual income: below CNY 100,000 = 1, CNY 100,000–200,000 = 2, CNY 200,000–400,000 = 3, CNY 400,000–600,000 = 4, above CNY 600,000 = 5. The square root of the AVE for each research variable is enclosed in diagonal parentheses. * *p* < 0.05, ** *p* < 0.01.

**Table 5:** Path Analysis Results.

	AI Service Awareness				AI Crafting		Proactive Service Performance	
	γ	*SE*	γ	*SE*	γ	*SE*	γ	*SE*
*Control variable*								
Gender	−0.10	0.08	−0.09	0.08	0.02	0.05	0.03	0.05
Age	0.06	0.05	0.06	0.05	−0.05	0.04	−0.02	0.05
Education	0.00	0.06	−0.01	0.06	−0.01	0.04	0.00	0.04
Organizational tenure	−0.01	0.01	−0.01	0.01	0.00	0.00	0.01	0.00
Family annual income	0.04	0.04	0.04	0.04	−0.03	0.03	0.03	0.03
*Predictor*								
Digital leadership	0.43 ***	0.04	−0.15	0.22	0.05	0.03	0.09 **	0.03
*Moderator*								
Public service motivation			−0.51 *	0.22				
Digital leadership × Public service motivation			0.16 *	0.06				
*Mediators*								
AI service awareness					0.20 ***	0.04	0.09	0.05
AI crafting							0.46 ***	0.07
*R* ^2^	0.35 ***		0.37 ***		0.19 ***		0.34 ***	

Note: *N* = 234. Unstandardized path coefficients were estimated; *SE* = Standard error; * *p* < 0.05, ** *p* < 0.01, *** *p* < 0.001.

## Data Availability

The data are not publicly available due to privacy restrictions in government organizations.

## Appendix A

**Table A1:** Item Description and Statistics.

Variables	Items	Factor Loading	CR	AVE
Digital leadership	My leader is very interested in using digital technologies and tools.	0.83	0.91	0.64
	My leader is an expert in digitization.	0.80		
	My leader is up to date with my digital knowledge.	0.84		
	My leader is actively driving the digital transformation of my organization	0.77		
	My leader fosters the enthusiasm of the organization members for digital transformation.	0.81		
	My leader has a comprehensive and clear understanding of the structures and processes required for the digital transformation of the organization.	0.75		
Public service motivation	Meaningful public service is very important to me.	0.81	0.85	0.53
	I am often reminded by daily events about how dependent we are on one another.	0.66		
	Making a difference in society means more to me than personal achievements.	0.77		
	I am prepared to make enormous sacrifices for the good of society.	0.63		
	I am not afraid to go to bat for the rights of others, even if it means I will be ridiculed.	0.77		
AI service awareness	I was very aware of AI used in my service work.	0.70	0.79	0.56
	I had a great deal of knowledge about AI used in my service work	0.80		
	I could quickly recall information I had received about AI in my service work.	0.74		
AI crafting	When working with AI, I introduce new approaches on my own to improve my work.	0.79	0.92	0.65
	When working with AI, I change minor work procedures which I think are not productive on my own.	0.84		
	When working with AI, I change the way I do my job on my own to make it easier for myself.	0.85		
	When working with AI, I rearrange the tasks or procedures of the work to collaborate with AI robots more effectively on my own.	0.79		
	When working with AI, I learn new knowledge and skills about AI robots on my own.	0.76		
	When working with AI, I try to find the ways to improve the interactions with AI robots on my own.	0.81		
Proactive service performance	I proactively share information with citizens to meet their needs.	0.78	0.86	0.56
	I anticipate issues or needs citizens might have and proactively develop solutions.	0.71		
	I actively create partnerships with other service representatives to better serve citizens.	0.80		
	I take the initiative to communicate client requirements to other service areas and collaborate in implementing solutions.	0.67		
	I proactively check with citizens to verify that their expectations have been met or exceeded.	0.77		
